# F-18 FDG PET/CT in staging and response assessment of primary cutaneous diffuse large B-cell lymphoma (leg type)

**DOI:** 10.1186/s41824-023-00174-5

**Published:** 2023-08-22

**Authors:** Deepanksha Datta, Rakesh Ramprakash Pandey, Rajesh Kumar, Rashim Sharma, Deepak Vedant

**Affiliations:** 1 Department of Nuclear Medicine, All India Institute of Medical Sciences, Jodhpur, India; 2 Department of Pathology and Lab Medicine, All India Institute of Medical Sciences, Jodhpur, India

**Keywords:** Primary cutaneous diffuse large B-cell lymphoma-leg type, PCDLBCL-LT, F-18 FDG PET/CT, Staging, Response assessment

## Abstract

Primary cutaneous Diffuse Large B-Cell Lymphoma-leg type (PCDLBCL-LT) is a rare subtype of cutaneous lymphomas, with high frequency of extra-cutaneous relapse and poor prognosis. We report a case of 70-year-old lady who was diagnosed with PCDLBCL-LT on biopsy and underwent a baseline F-18 FDG PET/CT, followed by interim and post-treatment PET/CTs. With this case report, we highlight the findings of F-18 FDG PET/CT in the staging of this cutaneous lymphoma, and also emphasize on its role in the response assessment.

## Introduction

A lymphoma is defined as primarily cutaneous when the lymphatic proliferation is limited to the skin with no evidence of extracutaneous disease at the time of diagnosis (Sokołowska-Wojdyło et al. 2015). Histo-pathological diagnosis along with immune-phenotyping is the gold standard for its diagnosis. The initial staging is done by assessing the percentage of the skin involvement, and number of nodes and extra-cutaneous sites involved by the disease (Kim et al. 2007). Primary cutaneous diffuse large B-cell lymphoma is a rare and aggressive subtype with a 5-year survival rate of 41–56% and higher extra-cutaneous involvement than other cutaneous forms (The 2018 update of the WHO-EORTC classification for primary cutaneous lymphomas | Blood | American Society of Hematology [Internet] 2018; Grange et al. 2014, 2007). F-18 FDG PET/CT is shown to be useful in staging assessment of PCDLBCL-LT (primary cutaneous diffuse large B-cell lymphoma); however, the literature is limited (Kumar et al. 2006; Dejust et al. 2020; Ni et al. 2016). With this case report, we highlight the utility of the F-18 FDG PET/CT in staging as well as response assessment and surveillance of this rare lymphoma.

## Case report

**Fig. 1:**
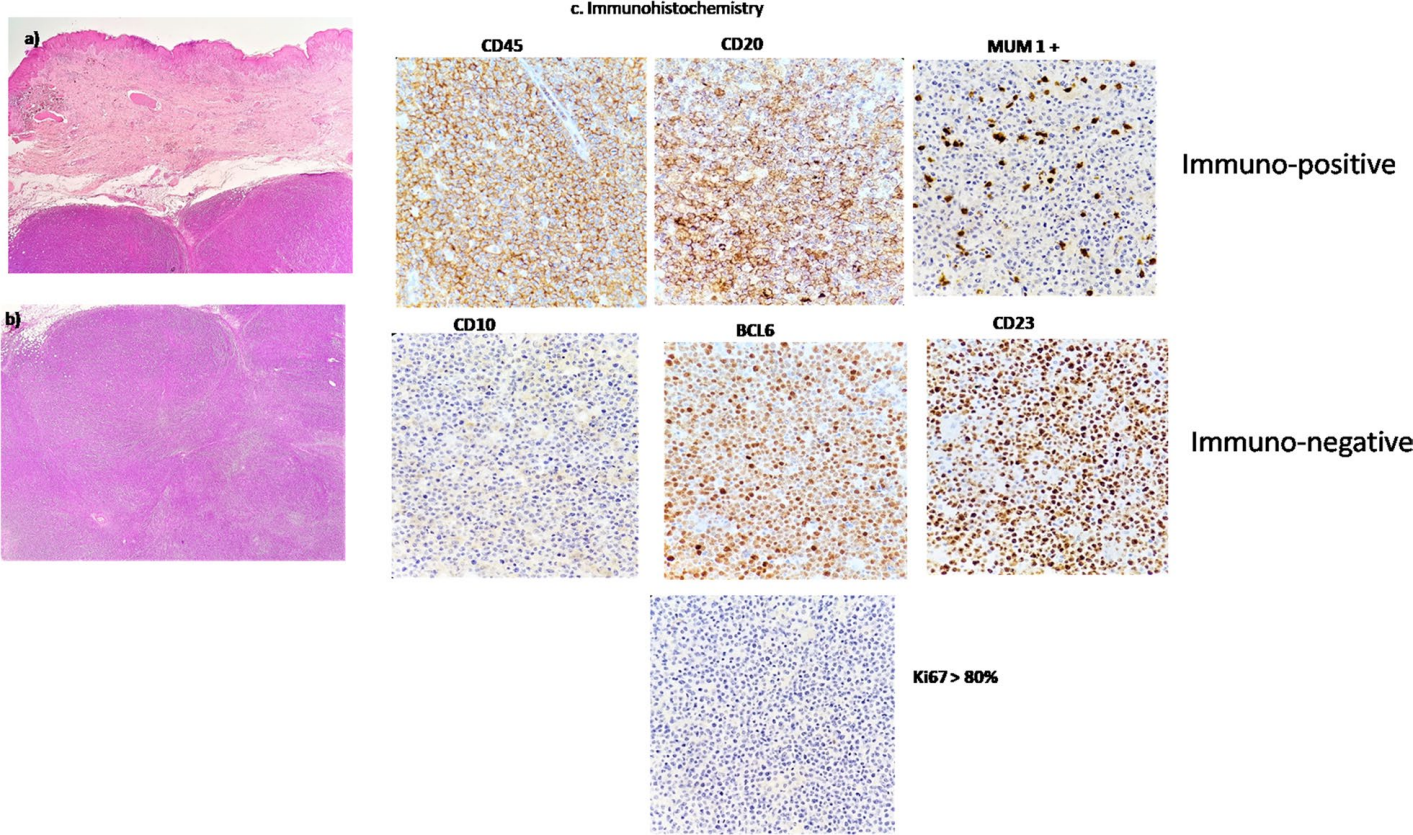
**a** Skin with tumour in subcutis arranged in nodular pattern (H&E, 2x), **b** Tumour cells forming nodules, confluent sheets and peritheliomatous arrangement, predominantly centroblasts and immunoblasts (H&E, 20x), **c** Immunohistochemistry was positive for CD45,CD20 and MUM 1(> 30% cells) and negative for CD10, BCL6 and CD23, with Ki67 index > 80%

**Fig. 2:**
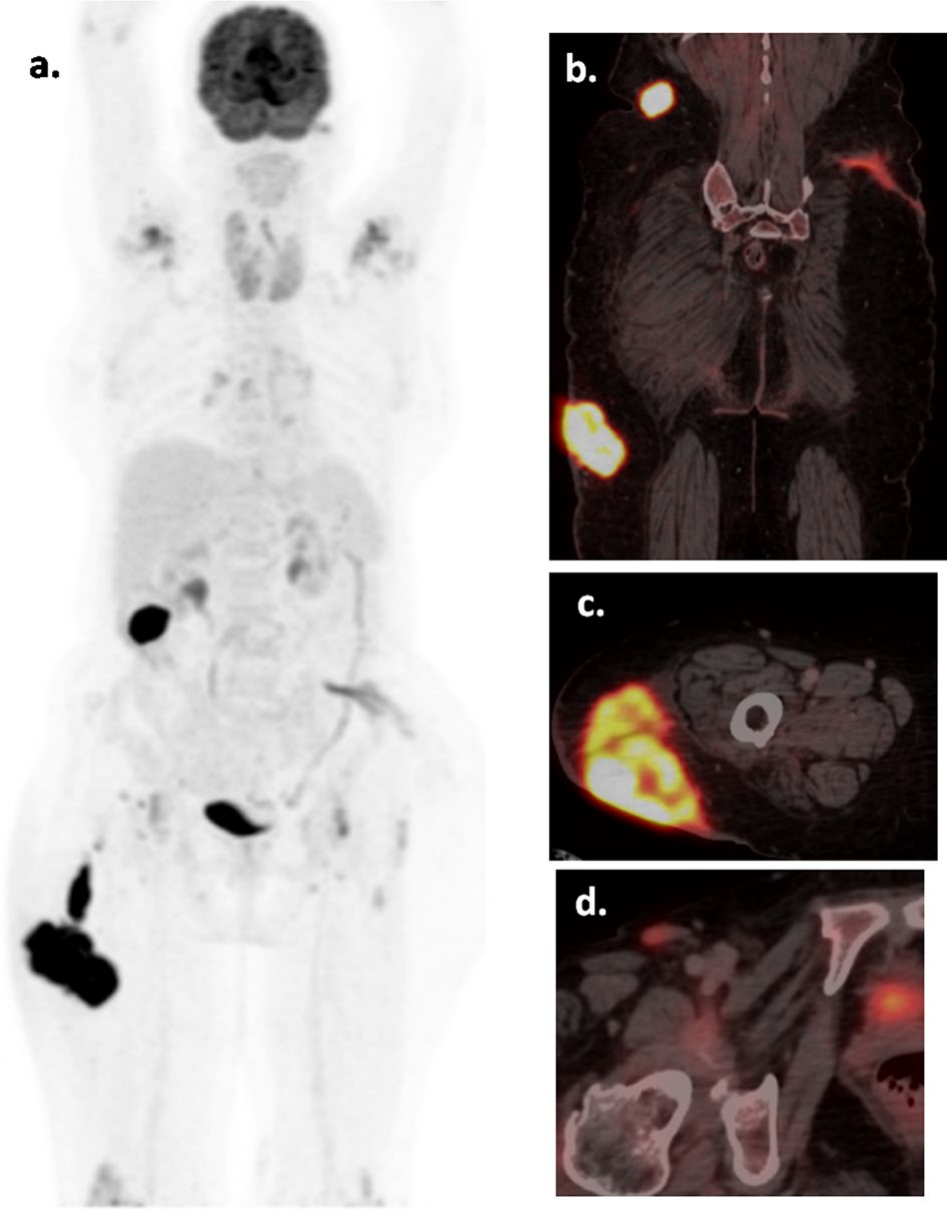
Baseline (staging) F-18 FDG PET/CT images, maximum intensity projection (MIP) image **a** showing multiple cutaneous and subcutaneous lesions in right lumbar–gluteal (**b**) and upper thigh (**c**) regions, along with prominent right inguinal lymph node (**d**)

**Fig. 3:**
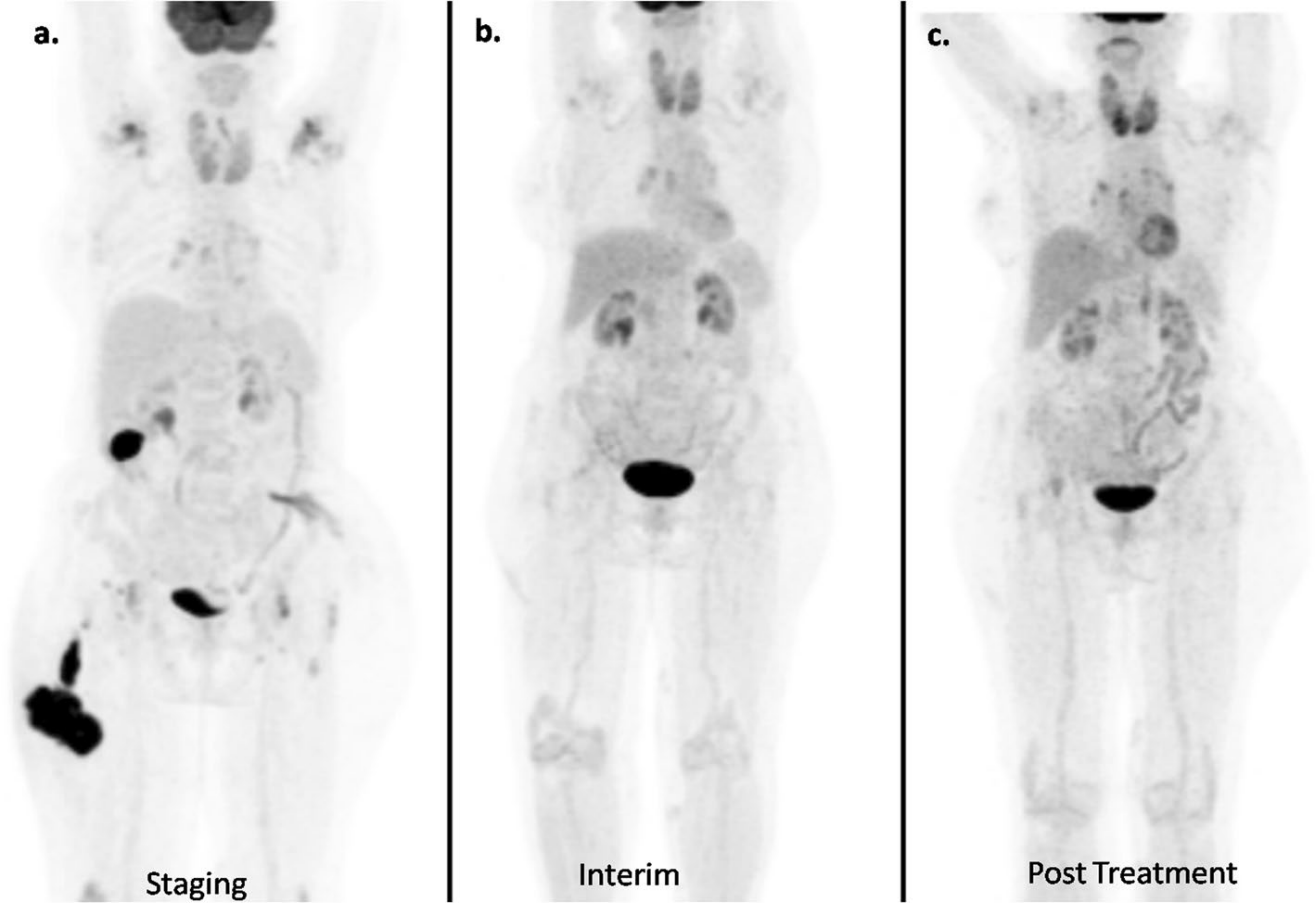
Comparison MIP images of F-18 FDG PET/CT in staging (**a**), interim (**b**) and post-treatment (**c**)

A 70-year-old lady had insidious onset of left gluteal swelling, followed by swelling in right gluteal region and right upper thigh. The excisional biopsy (Fig. 1a, b) from left gluteal swelling showed diffuse large B-cell lymphoma (DLBCL). Immunohistochemistry (Fig. 1c) was performed as per the Hans algorithm that showed immunopositivity for CD45, CD20, MUM 1 (> 30% cells), and immunonegativity for CD10, BCL6, and CD23. The Ki 67 labelling index was 80%. The baseline F-18 FDG PET/CT (Fig. 2) showed metabolically active (SUV max 12.6) multiple cutaneous—subcutaneous lesions in right gluteal, right lumbar and upper thigh regions along with few prominent right inguinal and external iliac (SUV max- 2.5) lymph nodes. Ancillary finding of multi-nodular goiter was noted. R-CHOP regime chemotherapy was started and an interim FDG PET/CT (Fig. 3b) done after 4 cycles of chemotherapy showed complete metabolic resolution of the cutaneous and subcutaneous lesions (Deauville score 2) as well as right inguino-pelvic lymph nodes (Deauville score 1). After completing 8 cycles of chemotherapy, the post-treatment PET/CT (Fig. 3c) showed persistent disease remission status.

## Discussion

Primary cutaneous diffuse large B-cell lymphoma was recognized as a unique entity by the WHO/EORTC classification system in 2005 (The 2018 update of the WHO-EORTC classification for primary cutaneous lymphomas | Blood | American Society of Hematology [Internet] 2018) and is characterized clinically by lesions preferentially in the leg. It is more common in the elderly female with frequent relapses and extra-cutaneous involvement (Grange et al. 2014, 2007; Samarghandi et al. 2015). Few reports have shown the intense FDG uptake in the cutaneous lesions (Dejust et al. 2020; Ni et al. 2016). However, there is limited literature assessing the role of PET/CT in response assessment owing to the rarity of the disease and is mainly extrapolated from the systemic DLBCL. In our patient, the staging PET/CT was done to evaluate the extent of cutaneous lesion and detect any extra-cutaneous disease. Metabolically active multiple cutaneous–subcutaneous lesions as well as the enlarged regional inguinal–pelvic lymph nodes were noted. The follow-up PET/CTs done during and after the completion of chemotherapy were evaluated using the Lugano criteria (Cashen et al. 2011). Both the interim and post-treatment PET/CT showed complete metabolic response. The patient is still on follow-up and suggested a surveillance PET/CT owing to the aggressive nature of the disease. With this case report, we emphasize on the utility of F-18 FDG PET/CT in staging as well as response assessment of this rare lymphoma.

## Data Availability

Not applicable.

## Abbreviations

PCDLBCL-LT: Primary cutaneous diffuse large B-cell lymphoma (leg type)
DLBCL: Diffuse large B-cell lymphoma
F-18 FDG: F-18 2-fluoro 2-deoxy D glucose
PET/CT: Positron emission tomography
WHO: Work Health Organization
EORTC: European Organization for Research and Treatment of Cancer
